# Photoprotection and Photostability of a New Lignin-Gelatin-*Baccharis antioquensis*-Based Hybrid Biomaterial

**DOI:** 10.3390/antiox10121904

**Published:** 2021-11-27

**Authors:** Juan C. Mejía-Giraldo, Juan C. Scaiano, Cecilia Gallardo-Cabrera, Miguel A. Puertas-Mejía

**Affiliations:** 1 Grupo de Investigación en Compuestos Funcionales, Facultad de Ciencias Exactas y Naturales, Universidad de Antioquia UdeA, Calle 70 No. 52-21, Medellín 050010, Colombia; juan.mejia8@udea.edu.co; 2 Grupo de Estabilidad de Medicamentos, Cosméticos y Alimentos, Facultad de Ciencias Farmacéuticas y Alimentarias, Universidad de Antioquia UdeA, Calle 70 No. 52-21, Medellín 050010, Colombia; cecilia.gallardo@udea.edu.co; 3 Department of Chemistry and Biomolecular Sciences, University of Ottawa, Ottawa, ON K1N 6N5, Canada; titoscaiano@mac.com

**Keywords:** *Baccharis antioquensis*, gelatin, hybrid biomaterial, lignin, photoprotection, photostability

## Abstract

The aim of this study was to develop a new hybrid biomaterial that could photo-stabilize and improve the photoprotective capacity of a *Baccharis antioquensis* extract. Different combinations of lignin/gelatin/natural extract were applied to prepare hybrid biomaterial nanoparticles (NPs), which were then incorporated into an emulsion. The in vitro photoprotection and photostability were evaluated. The methanolic extract showed high phenolic content (646.4 ± 9.5 mg GAE/g dry extract) and a DPPH radical assay revealed that the antiradical capacity of the extract (0.13 to 0.05 g extract/mmol DPPH) was even better than that of BHT. The particle size of the hybrid biomaterial ranged from 100 to 255 nm; a polydispersity index (PdI) between 0.416 and 0.788 is suitable for topical use in dermocosmetic products. The loading capacity of the extract ranged from 27.0 to 44.5%, and the nanoparticles (NPs) showed electrostatic stability in accordance with the zeta potential value. We found that the formulation based on lignin: extract (1:1 ratio) and gelatin: lignin: extract (0.5:0.5:1 ratio) demonstrated photoprotection qualities with a sun protection factor (SPF) ranging from 9.4 to 22.6. In addition, all the hybrid NP-formulations were time-stable with %SPFeff and %UVAPFeff greater than 80% after exposure to 2 h of radiation. These results suggest that the hybrid biopolymer-natural extract improved the photoprotection and photostability properties, as well as the antiradical capacity, of the *B. antioquensis* extract, and may be useful for trapping high polyphenol content from natural extracts, with potential application in cosmeceutical formulations.

## 1. Introduction

Currently, there is a limited number of organic synthetic UV filters, which have been used as active ingredients in sunscreens. Some of them can promote the formation of reactive intermediates, such as free radicals, which compromises their safety and efficacy [1]. Furthermore, their photolysis products could generate photo-contact dermatitis [2]. Inorganic filters, e.g., titanium dioxide (TiO_2_) or zinc oxide (ZnO), reflect UV radiation and are good broad-spectrum protectors, but their particle size is the main disadvantage in their use in the preparation of homogeneous dermo-cosmetic formulations [3]. Furthermore, TiO_2_ nanoparticles are involved in reactive oxygen species (ROS) production through exposure to UVR [4,5].

Natural products are gaining an increasingly important role as alternative sources of chemical compounds with UV absorption capacity [6,7]. Enriched extracts containing an effective mixture of compounds are an alternative method through which to produce broad-spectrum sunscreens. However, these organic filters could be photodegradable, and some alternatives to overcome this drawback have been developed, such as encapsulated systems that protect them from adverse conditions, including exposure to solar ultraviolet radiation (UVR) [8]. Furthermore, encapsulation techniques improve the biological properties of natural products [9,10]. The aim of this study was to develop novel bioparticles using a combination of natural polymers that can encapsulate natural extracts while preserving their photochemical properties.

*Baccharis* is a genus of the Asteraceae family from tropical and subtropical areas of America. It is one of the most important genus composed of flora in Colombia [11]. *Baccharis antioquensis* is an endemic plant of Colombia; it is found between 2500 and 3000 m.a.s.l. Our previous research showed that the methanolic extract of *B. antioquensis* demonstrated excellent photoprotection activity and a high polyphenolic composition. Furthermore, we reported the presence of three glycosides of quercetin (quercetin 3-O-xilopyranosyl-(1→6)-glucopyranoside, quercetin 3-O-rhamnopyranosyl-(1→6)-glucopyranoside and quercetin 3-O-(4‴-O-caffeoyl)-rhamnopyranosyl-(1→6)-galactopyranoside), a kaempferol glycoside (kaempferol 3-O-rhamnopyranosyl-(1→6)-glucopyranoside), and a derivative of caffeic acid (3-O-caffeoylquinic acid) [12].

Gelatin is a biodegradable and biocompatible natural polymer obtained from collagen, which has previously been used in medicinal and pharmaceutical products [13,14,15]. In addition to its mucoadhesive properties, this polymer could enhance the effectiveness of topic formulations [16]. Lignin, for its part, is the second most abundant biopolymer on Earth. It is mainly obtained as a waste product from the pulp and paper industries. The multiple phenolic functions present in lignin are responsible for its high antioxidant activity and UV spectroscopy properties [17]. Recently, Qian et al. [18] reported that lignin is capable of enhancing the SPF value of commercial sunscreens. Here, we present our efforts to obtain hybrid material based on lignin/gelatin/natural extract NPs, with biodegradable and photostability characteristics that make them suitable for potential applications in cosmetic formulations.

## 2. Materials and Methods

### 2.1. Materials and Instrumentation

The lignin (low sulfonate content), sorbitan monooleate (Span 80), 2,2-Diphenyl-2-picrylhydrazyl (DPPH) stable radical and gallic acid were purchased from Sigma-Aldrich (St. Louis, MO, USA). The methanol, ethanol, hexane, Folin–Ciocalteu phenol reagent and sodium carbonate were obtained from Merck Chemical Supplies (Damstadt, Germany). The butylated hydroxy toluene (BHT) was purchased from Alfa Aesar (Ward Hill, MA, USA). The gelatin USP (Granular 100 bloom) and mineral oil were obtained from Fisher Scientific Company (Hampton, NH, USA). The lanolin, cetyl alcohol, glyceryl monostearate, stearic acid, sorbitol, and triethanolamine, were purchased from JM. Chemicals. The polymethylmethacrylate (PMMA) plates, Helioplate HD6, were from Labsphere (North Sutton, NH, USA).

All the spectrophotometric data were obtained using either one of the following: a Thermo Scientific Evolution 60S UV-Visible spectrophotometer (Shanghai, China); a Cary 60, Agilent Technologies UV-Vis spectrophotometer (Santa Clara, CA, USA); or a UV Transmittance Analyzer UV-2000S, Labsphere (North Sutton, NH, USA). The homogenization of the formulations was performed with a Kinematica Polytron PLU-2-110 homogenizer (Fisher Scientific, Hampton, NH, USA). The sample centrifugations were carried out in the Eppendorf Centrifuge 5804 R. (Eppendorf AG, Hamburg, Germany) Scanning Electron Microscopy (SEM) imaging was performed by scanning the coated samples in powder and grids with a Jeol JSM-1600 SE Microscope (Tokyo, Japan). The size, polydispersity index (PdI), and zeta potential of each NP sample were determined using the Zetasizer Nanoseries Nano-ZS from Malvern (Malvern, UK). A solar simulator apparatus equipped with a xenon arc lamp (1500 W) and special UV glass filters cutting off radiation below 290 nm were used.

### 2.2. Extraction Procedure

The plant material was collected in September 2013 in Llanos de Cuivá, Yarumal, Antioquia, Colombia, at 2730 m above sea level (at geographic coordinates of 6°49′50.6″ N; 75°29′29.9″ W). A voucher specimen (HUA194796) was deposited in the herbarium of the University of Antioquia, Colombia (Contract for Access to Genetic Resources and their Derivative Products N° 252, Resolution 0399-Ministerio de Ambiente y Desarrollo Sostenible, Colombia). The extraction procedure was performed according to the method described by Mejia-Giraldo et al. [12], with slight modifications. Fresh vegetal materials (leaves) were dried at room temperature protected from natural and artificial light. Next, dry vegetal materials (DVM) were crushed using an electric grinder (IKA, A11 basic S1) (Wilmington, NC, USA). Briefly, 180 g of crushed DVM were degreased using 1.8 L of hexane and magnetic stirring for 6 h (ca. 25 °C). Next, the defatted material was subjected to four successive extractions using methanol at room temperature (ca. 25 °C) with magnetic stirring for 24 h. Subsequently, the extract was filtered and treated with 180 g activated carbon, and it was again filtered. Finally, the crude extracts obtained were concentrated to dryness in a rotary evaporator (Wilmington, NC, USA) (IKA, RV10 basic) at 40 ± 2 °C. According to the United States Pharmacopeia (USP) [19], methanol is Class 2, so the latter procedure was sufficient to remove methanol from the extracts and could be considered acceptable for cosmetic applications. Furthermore, in previous research, we reported low cytotoxicity in a U937 cell model and the low hemolytic capacity of the dry extract obtained with this solvent [12].

### 2.3. Antiradical Screening Assays

#### 2.3.1. Total Phenolic Content

The total phenolic content (TPC) of the sample was measured using a modified colorimetric Folin–Ciocalteu [20] method with some modifications. Briefly, 10 µL of extract solution and 615 µL of deionized water were added to a test tube. Subsequently, 125 µL of Folin-Ciocalteu reagent were added to the solution and allowed to react for 5 min. Next, 1250 µL of sodium carbonate solution (20% *w*/*v*) were added into the test tubes and mixed. The absorbance was read at 760 nm using an Evolution 60S spectrophotometer (Thermo Fisher Scientific, Inc., Shanghai, China). The results are expressed as milligrams of gallic acid equivalents per g dry extract (mg GAE/g DE) (y = 0.1199412x + 2.243652 × 10^−2^, r = 0.99993), where y = absorbance and x = concentration of gallic acid.

#### 2.3.2. Antiradical Activity-DPPH Assay

Different concentrations of extracts were estimated according to the method described by Mejia-Giraldo et al. [12]. The effective relative concentration (EC_50_) at which 50% of DPPH was removed was expressed as mg of dry extract/mmole DPPH radical, based on Equation (1).

EC50 = Concentration of sample at steady state/initial concentration of DPPH(1)

The initial concentration of DPPH (100 µmole/L) in the reaction system was calculated in relation to a curve (y = 1.146 × 10^−2^x − 4.192 × 10^−3^; r = 0.9999) at 514 nm, where y = absorbance and x = concentration of DPPH. All the spectrophotometric data were obtained using a Thermo Scientific Evolution 60S UV-Visible spectrophotometer (Shanghai, China). BHT standard was used as the positive control.

### 2.4. Hybrid Material: Synthesis and Characterization

#### 2.4.1. Preparation of Gelatin and Lignin Nanoparticles

The NPs were prepared through an emulsification process according to the methods reported by Lim [21] and Patel [22], with some modifications. The NPs of each polymer and mixtures thereof containing the extract in various proportions were prepared following the composition described in Table 1. The starting materials (gelatin, lignin, and extract) were dissolved in distilled water. The gelatin was heated at 40 °C in order to facilitate its dissolution. Subsequently, an emulsion was prepared by adding the aqueous solution dropwise to 40 g of mineral oil containing Span 80 (1%, *w*/*w*) as the emulsifying agent, under stirring at 5000 rpm, using a homogenizer. Once the emulsion was formed, the crosslinking process was induced by heating using a water bath at 40 °C for 24 h. Subsequently, the emulsion was centrifuged at 3000 rpm for 20 min and three layers were obtained (upper layer: mineral oil; middle layer: particles; bottom layer: aqueous phase). Thereafter, the oil and aqueous phases were removed, and the NPs were separated and washed twice with 10 mL of distilled water and four times with 20 mL of hexane. After each washing, the particle suspension was centrifuged at 3000 rpm for 10 min and the supernatant and the lower phase were discarded. Afterwards, the particles were dried at 40 °C in an oven for one week and then washed with 10 mL of hexane. Finally, the particles were dried again at 40 °C in the oven for one week. The hybrid NPs procedure and characterization assays were performed in triplicate.

#### 2.4.2. Characterization of Gelatin and Lignin Nanoparticles

The samples for the Scanning Electron Microscopy (SEM) study were prepared by lightly sprinkling the formulation on a double adhesive tape stuck to an aluminum stub. The samples were then coated with gold to a thickness of ca. 30 nm under argon atmosphere using a gold sputter module in a high-vacuum evaporator. Furthermore, the nanoparticle samples in aqueous dispersion were placed on copper grids with a carbon film.

The size, polydispersity index (PdI) and zeta potential of each NP sample were determined using dispersions of each NP in ethanol at concentration of 0.1 mg/mL and placed in a cuvette at 25 ± 2.0 °C. Three samples were prepared and ten readings of each sample were performed. The data obtained were evaluated through Zetasizer software (Malvern, UK), using the size distribution by number. Finally, dispersions of each nanoparticle and the starting polymers (gelatin and lignin) in distilled water at 0.025 mg/mL were prepared and the UV absorption spectra were measured within the 250 to 400 nm range.

#### 2.4.3. Loading Capacity (%LC) and Entrapment Efficiency (%EE) of Hybrid NPs

The %LC and %EE were determined by measuring the concentration of free extract in the dispersion medium. A total of 12.5 mg of NPs was dispersed in 25 mL of distilled water and subjected to ultrasonic agitation for 30 min at ca. 40 °C. Next, the dispersion was centrifuged at 3000 rpm for 10 min, and the supernatant was filtered through a 0.20 µm membrane. Finally, the extract content in the solution (250 µL of supernatant in 5 mL of distilled water) was quantified by the absorption at 325 nm, according to the maximum absorbance of the polyphenol compounds from a curve (y = 21.329x − 0.0008, R² = 0.9993; where y = absorbance and x = concentration of polyphenol extract of *B. antioquensis*).

The %LC was expressed as mg of extract per 100 mg of NP and the %EE was calculated according to Equations (2) and (3), respectively [23]:

% Loading capacity (%LC) = [W_e_/W_NPs_] × 100 (2)

% Entrapment efficiency (%EE) = [W_e_/W_a_] × 100 (3)

where W_NPs_ is the amount (mg) of NPs in the assay; We is the amount (mg) of extract encapsulated in the NPs and Wa is the amount (mg) of extract added in the preparation of NPs.

### 2.5. Preparation of Topical Emulsion to Be Used in the Evaluation of the NPs’ Photostability and Photoprotection

#### 2.5.1. Preparation of Topical Emulsion

Different emulsions (oil-in-water) containing each of the six types of NPs were prepared as shown in Table 2. Briefly, phase A (oil phase) was heated up to ca. 70–80 °C and the NPs were added and mixed with a homogenizer at 2000 rpm. Afterward, the phase B (water phase) was added slowly to phase A while stirring at 2000 rpm for 5 min, then the system was cooled down to room temperature. Table 3 shows the NP composition of each formulation. The control formulations were also prepared for evaluation: emulsion without extract, emulsion with 5% *w*/*w* lignin (negative controls), and commercial sunscreen with SPF 25 (positive control).

#### 2.5.2. In Vitro Determination of Photoprotective Capacity

The photoprotective capacity was evaluated in vitro by diffuse reflectance spectroscopy with an integrated sphere. The sunscreen formulation was accurately applied (0.75 mg/cm^2^) to roughen the polymethyl methacrylate (PMMA) plates and distributed uniformly over the whole surface using a cot-coated finger. Next, the film was left to equilibrate in a dark place at room temperature (25 ± 2 °C) for 15 min [24,25,26]. UV transmission measurements (from 290 to 400 nm) were performed using a spectrophotometer equipped with an integrating sphere. In vitro photoprotection efficacy was calculated according to the following parameters: UVB efficacy by estimating sun protection factor (SPF); and UVA efficacy by UVAPF, UVA/UVB ratio and critical wavelength (λ_c_). Three plates were prepared by formulation and nine different points per plate were measured for each sample.

#### 2.5.3. Photostability of Sunscreen Formulations

The photostability study was carried out using the method used by Jarzycka et al. [24]. The plates, prepared according to the steps outlined in the previous section, were irradiated for 2 h (taking measurements every 30 min) under simulated solar conditions. The light source emission was maintained at 650 W/m^2^ in accordance with the global solar spectral irradiance. Before and after irradiation, all the characteristic parameters of the photoprotection of the formulations (SPF, UVAPF, UVA/UVB ratio and critical wavelength (λ_c_)) were measured in vitro. The degree of photostability was expressed as the percentage effectiveness from SPF in vitro (% SPF_eff_) and UVAPF (%UVAPF_eff_) which were calculated according to Equations (4) and (5), respectively [27,28]. Three plates were prepared by formulation and nine different points per plate were measured for each sample.

%SPF_eff_ = (SPF_in vitro_ after irradiation/SPF_in vitro_ before irradiation) × 100 (4)

%UVAPF_eff_ = (UVAPF_in vitro_ after irradiation/UVAPF_in vitro_ before irradiation) × 100 (5)

### 2.6. Statistical Analysis

The results were expressed as the means ± SD. All the data were analyzed by one-way analysis of variance (ANOVA) and followed by Tukey tests when appropriate, using R Development Core Team (2011) R: A Language and Environment for Statistical Computing. The *p* values that were lower than 0.05 (*p* < 0.05) were considered significant [29].

## 3. Results and Discussion

### 3.1. Extraction Yield, TPC, and Antiradical Activity

According to the proposed modification by Mejía-Giraldo et al. [12] to the extraction of *B. antioquensis*, four successive extractions were carried out. As a result, an increase in the yield percentage from 27.65 ± 1.28 to 35.5 ± 2.1% was obtained; the TPC increased from 277.3 ± 7.6 to 646.4 ± 9.5 mg GAE per g dry extract and the antiradical capacity was improved from 0.13 ± 0.01 to 0.05 ± 0.01 g extract mmol^−1^ DPPH, which was even better than the antiradical capacity of BHT (0.11 ± 0.01 g of antioxidant mmol^−1^ DPPH). This was related to the successive extractions, which improved the extraction efficiency of the polyphenol.

In our previous research [12], we evidenced the antioxidant capacity of *B. antioquensis* extracts through a DPPH radical assay as well as through the oxidation of methyl linoleate, considering that linoleic acid is one of the main components of lipid membranes within cells. A good correlation between both results was found. These findings are relevant because the UVR produces reactive oxygen species (ROS), which can oxidize proteins, lipids, and DNA bases such as 8-dihydroxy-2-deoxyguanosine, and therefore cause cancer [30,31]. Consequently, the use of filters with antioxidant activity in a photoprotective formulation is an effective approach to prevent harmful effects of UVR in skin [32,33,34].

### 3.2. Characterization of Lignin-Gelatin-Extract Nanoparticles

**Figure 1:**
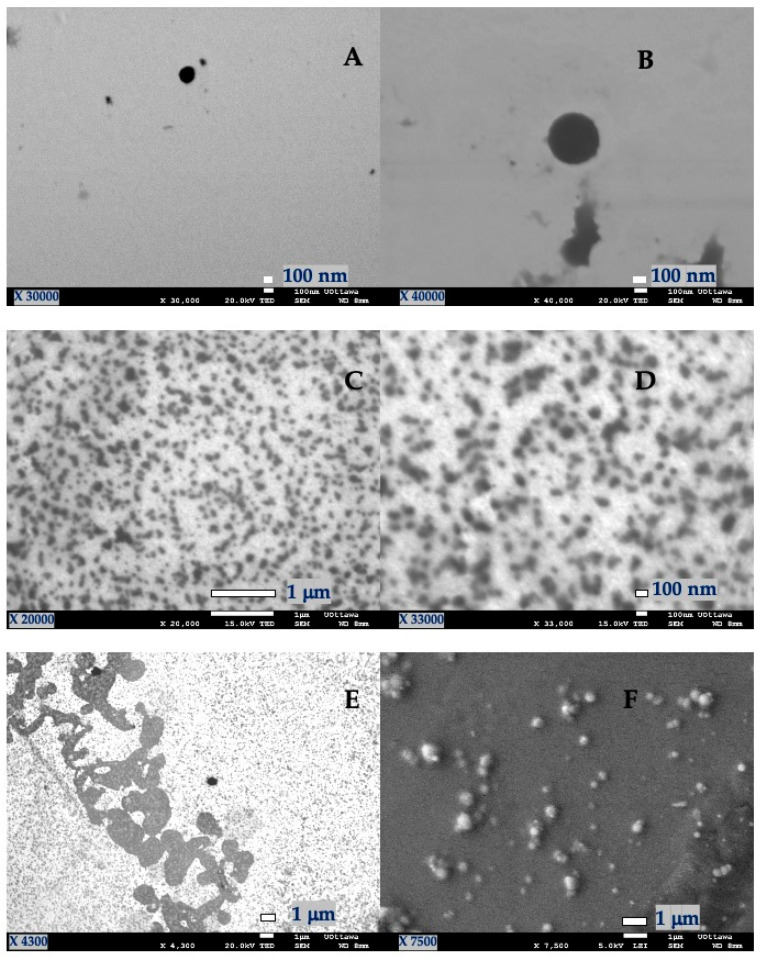
SEM micrographs of nanoparticles. (**A**,**B**). G-E (1:1); (**C**,**D**). L-E (1:1); (**E**,**F**). G-L-E (0.5:0.5:1).

The SEM images (Figure 1) show that spherical NPs were obtained in all cases. The starting materials of the NPs (lignin and gelatin) provided a hydrophilic environment. Particle aggregation observed from the SEM images and DLS analysis indicated an inverse relationship between the mean particle diameter and the *B. antioquensis* extract concentration. Since the *B. antioquensis* extract also exhibited a hydrophilic character, it could be entrapped into the NPs and protected against the degradation caused by UVR, thereby preserving its biological activities, such as photoprotective and antioxidant capacity. However, more work is needed to assess its performance and overall synthesis.

In the DLS analyses, the particle size ranged from 99 to 254 nm and the PdI varied from 0.416 to 0.788 (Table 4). Among the NPs evaluated, those with the composition G:E (1:0.5 ratio) formed the biggest particles, which were estimated at 253 nm. These results are in agreement with those odd Jabar et al. and Gaur et al. [35,36], who associated the high viscosity of the solution with the formation of bigger droplets that lead to the formation of larger nanoparticles. This effect was also observed in G-L-E NPs (Table 4).

Although the PdI values indicate a broad size distribution, the polydisperse particle dispersions obtained were suitable and did not affect either the performance or the sensorial characteristics for topical application [37]. All the NPs remained in suspension; this was likely due to their electrostatic stability, according to the Zeta potential values obtained [38].

The %LC ranged from 27.0 to 44.5%, which is considered a good outcome for NPs based on biopolymer raw material. According to the preparation method, subsequent washing process of the NPs and the water solubility of the polyphenolic compounds of *B. antioquensis*, we suggest that the latter are distributed inside the NPs and not only adsorbed on the surface. Regarding the NPs with a 1:1 polymer-extract ratio, the loading capacity was between 43.2 ± 1.5 and 44.5 ± 1.4%. The NPs with a 1:0.5 polymer-extract ratio exhibited a decrease in the loading (27.0 ± 0.9–33.9 ± 1.0%), which was due to the lower concentration of extract in the solution. Furthermore, the measurement of the %EE in the NPs with a 1:0.5 ratio was higher (60.3 ± 7.6–65.2 ± 5.4%) compared to the values for the 1:1 ratio (19.4 ± 0.6 and 52.6 ± 0.2%) (Table 4). This could have been associated with the NPs’ saturation, which would have prevented the extract from adsorbing into the interstices of the polymer matrix, remaining on the outside, and subsequently being eliminated in the washing process. These results showed that Ns formation depends on factors such as the concentration of gelatin and lignin, as well as the biopolymer to *B. antioquensis* extract ratio. The NPs synthetized in this work can be useful for trapping enriched polyphenol natural extracts with potential applications in topical formulations.

### 3.3. Photoprotective Capacity and Photostability of Hybrid NPs

**Figure 2:**
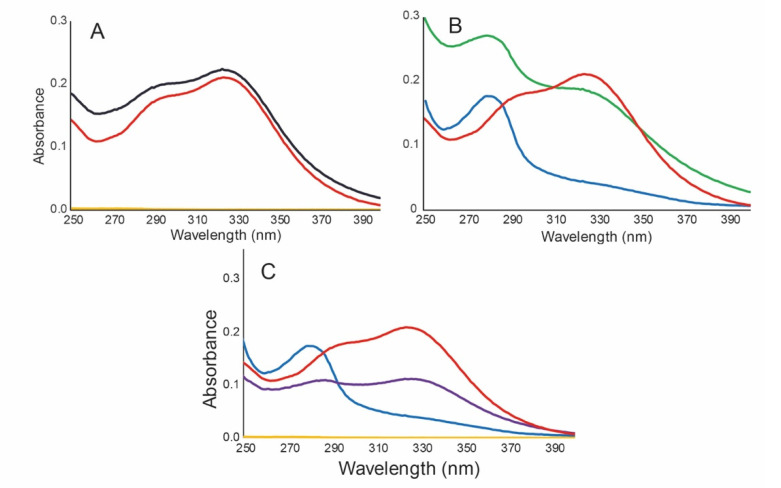
UV spectra of NPs and polymers 0.025 mg/mL in milliQ water. (**A**). Gelatin (yellow), *B. antioquensis* extract (red), and NP G-E (1:1) (black). (**B**). Lignin (blue), *B. antioquensis* extract (red) and NP L-E (1:1) (green). (**C**). Gelatin (yellow), *B. antioquensis* extract (red), Lignin (blue) and NP G-L-E (0.5:0.5:1) (purple).

UV filters are contained in several types of vehicles, such as silica, chitosan and hyaluronic acid microparticles [39,40], each one with peculiar characteristics. In our case, we used biopolymer-based NPs and the effect of the NPs on the UVA-UVB protection effectiveness was observed (Figure 2, Table 5).

Figure 2A shows that the *B. antioquensis* extract was exclusively responsible for the UVA-UVB absorption in NP G-E (1:1); conversely, the gelatin (yellow line) had no absorption over this range. On the other hand, the lignin (blue line Figure 2B,C) showed an absorption maximum wavelength at 280 nm. This absorption was also detected in the NP L-E (green line Figure 2B) and G-L-E (purple line Figure 2C) spectra. In both cases, the lignin exhibited an additive effect on the spectrum of the extract from 280 to 330 nm and, consequently, the photoprotection properties of the extract were substantially increased (Table 5).

The formulations named 5 (G-E NP 1:0.5 ratio), 6 (L-E 1:0.5 ratio), and 7 (G-L-E 0.5:0.5:0.5 ratio), exhibited very intense brown coloration, low spreadability, and high stickiness, in accordance with the high polymer content (Table 3). These results could affect consumer acceptance (due to the color of the formulations), and could affect the application of the product on the skin, since it was difficult to spread them on the PMMA plates. Instead, the formulations named 2 (G-E 1:1 ratio), 3 (L-E 1:1 ratio), and 4 (G-L-E 0.5:0.5:1 ratio) exhibited adequate sensorial and photoprotection qualities. Furthermore, the NPs with the 1:0.5 polymer-extract ratio demonstrated lower SPF values, between 9.4 and 14.2, and a UVAPF between 5.0 and 7.3; with respect to the formulations with the NPs with a 1:1 polymer-extract ratio, the SPF values were between 13.9 and 22.6 and the UVAPF between 6.0 and 8.7. This was expected because of the low loading capacity obtained with the NPs with the 1:0.5 ratio (27.0 and 30.2% *w*/*w*) and the polar characteristics of the biopolymers (gelatin and lignin), which could affect the selective encapsulation of the polar compounds present in the *B. antioquensis* extract (mainly glucosides). On the other hand, it was evidenced that in the formulations of lignin NPs, this contributed significantly to the UVA-UVB photoprotection properties, since higher values of SPF and UVAPF were obtained than in those in which they were not present. In addition, the photoprotective effect of lignin was observed in the negative control formulation (Emulsion + Lignin (5% *w*/*w*)) with SPF = 3.3 ± 0.3 and UVAPF = 2.0 ± 0.3 (Table 5).

Finally, the critical wavelength (λ_c_), UVA/UVB ratio, and sun protection factor of all the formulations were in accordance with the COLIPA and FDA regulation parameters. All the λ_c_ values obtained during the evaluation were above 370 nm and the values of the UVAPF were higher than 1/3 of the SPF, which satisfied the requirements for broad-spectrum UVA-UVB protection. In addition, the UVA/UVB ratio ranged from 0.6 to 0.8 (equivalent to three stars), which is considered high protection level in the UVA, according to the Boots Star Rating system [41]. Additionally, both the λ_c_ values and the UVA/UVB ratio in all the formulations did not change significantly in the photostability study and were never below 370 nm or 0.6, respectively (Table 5).

**Figure 3:**
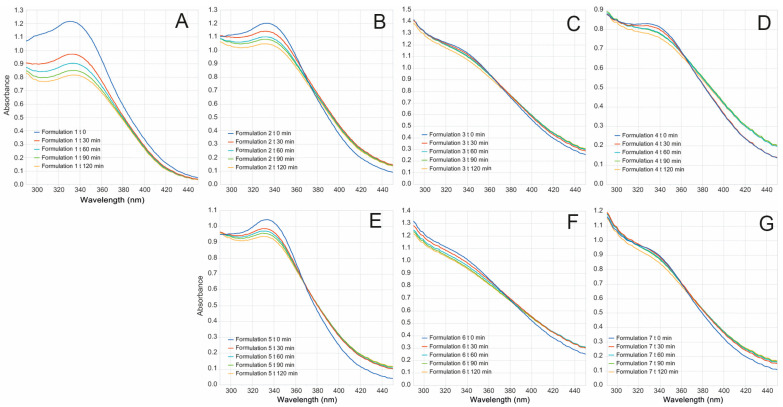
Photo-stability of *B. antioquensis* extract and NPs. UV-vis spectra before (blue) and after 30 min (red), 60 min (light blue), 90 min (green), and 120 min (yellow) under UVA-UVB irradiation. Average measurement of formulation: (**A**). F1; (**B**). F2; (**C**). F3; (**D**). F4; (**E**). F5; (**F**). F6; (**G**). F7 (See Table 5 for formulation details).

A product is considered photostable when the %SPF eff and %UVAPFeff are at least 80% after exposure to radiation. In this sense, the photostability assays showed that all the hybrid NP formulations were time-stable with %SPFeff and %UVAPFeff greater than 80% after exposure to 2 h of radiation (Figure 3, Table 5). The (1:1 ratio) NP-lignin-extract formulation exhibited higher photoprotection and photostability parameters in comparison to the free-NPs formulation (improved photostability between 38 and 47%). These outcomes may imply that encapsulation methodology enhances the photostability properties of the free-NP *B. antioquensis* natural extract, as reported previously [12].

## 4. Conclusions

An interesting spherical and relatively low polydispersed hybrid polymer nanoparticle composed of gelatin-lignin and extract of *B. antioquensis* were synthetized. The systems evaluated, consisting of a mixture of different biopolymers, and the data showed that all of them could help to obtain emulsion with a satisfactory overall photostability. Moreover, the formulation containing lignin: extract NPs improved the photoprotection capacity of the single extract at a level very close to that of commercial sunscreens, and the photostability was remarkably upgraded to 38–47% in the *B. antioquensis* extract cream formulation (F1). Finally, these NPs can be useful for trapping high contents of polyphenol from natural extracts, with a prospective application in pharmaceutical and cosmetic formulations. To the best of our knowledge, this is the first report on nanoparticles based on *B. antioquensis* extract and biopolymers that has improved the photoprotective properties of the evaluated raw material.

## Figures and Tables

**Table 1:** Polymer-extract composition ratio of nanoparticles as prepared.

Composition	Gelatin	Lignin	Extract
Gelatin-Extract (G-E)	1	-	1
Lignin-Extract (L-E)	-	1	1
Gelatin-Lignin-Extract (G-L-E)	0.5	0.5	1
Gelatin-Extract (G-E)	1	-	0.5
Lignin-Extract (L-E)	-	1	0.5
Gelatin-Lignin-Extract (G-L-E)	0.5	0.5	0.5

**Table 2:** Composition of the sunscreen emulsion formulations.

Components	% Formulation (*w*/*w*)
*Phase A* (*oil phase*):	
Lanolin	4.5
Cetyl alcohol	2.0
Glyceryl monostearate	3.0
Stearic acid	2.0
Nanoparticles	X = amount of NPs equivalent to 10% (*w*/*w*) of dry extract (See Table 3)
*Phase B* (*aqueous phase*):	
Sorbitol	5.0
Triethanolamine	1.0
Water	Sufficient quantity to 100%.

**Table 3:** Amount of nanoparticles in each formulation.

Formulation	Composition (Ratio)	% (*w*/*w*) *
F1	*B. antioquensis* extract	10.0 †
F2	G-E (1:1)	23.1
F3	L-E (1:1)	23.1
F4	G-L-E (0.5:0.5:1)	22.5
F5	G-E (1:0.5)	37.0
F6	L-E (1:0.5)	29.5
F7	G-L-E (0.5:0.5:0.5)	33.1
Negative control	Active free emulsion	-
Negative control	Emulsion + Lignin (5% *w*/*w*)	-
Positive control	Commercial sunscreen (CSS) SPF 25	-

* Equivalent to 10% (*w*/*w*) of dry extract in each formulation. † Dry extract.

**Table 4:** Physicochemical characterization of the hybrid biomaterial nanoparticles.

NPs	Mean Size, nm	PdI	ς Potential, mV	Yield * %	Loading Capacity% †	Entrapment Efficiency% ‡
G-E (1:1)	107 ± 38	0.653	−39.3 ± 2.7	60.9 ± 2.0	43.2 ± 2.3	52.6 ± 0.2
L-E (1:1)	99 ± 32	0.416	−45.5 ± 3.2	46.9 ± 2.1	43.2 ± 1.5	40.5 ± 0.3
G-L-E (0.5:0.5:1)	109 ± 39	0.788	−50.3 ± 1.2	21.8 ± 3.7	44.5 ± 1.4	19.4 ± 0.6
G-E (1:0.5)	253 ± 39	0.503	−38.6 ± 0.5	78.4 ± 3.2	27.0 ± 0.9	60.3 ± 7.6
L-E (1:0.5)	134 ± 22	0.548	−62.4 ± 0.9	72.0 ± 1.1	33.9 ± 1.0	65.2 ± 5.4
G-L-E (0.5:0.5:0.5)	167 ± 47	0.592	−54.2 ± 0.7	69.9 ± 1.4	30.2 ± 1.3	63.1 ± 3.6

* mg NP per 100 mg of reagents. † mg extract per 100 mg of NP. ‡ mg nano-encapsulated extract per 100 mg of extract added. G: Gelatin. L: Lignin. E: *B. antioquensis* extract. NP: Nanoparticles.

**Table 5:** In vitro photoprotective capacity and photostability of hybrid polymer-extract nanoparticles (NPs).

	SPF †		UVAPF ‡	λ_c_	UVA/UVB	
Active free emulsion	0.93 ± 0.01		0	-	-	
Emulsion + lignin 5%	3.33 ± 0.31		2	376	0.53	
CSS * SPF 25	26.18 ± 1.11		3.0 ± 0.0	356	0.43	
Time (min)	0		30	60	90	120
Emulsion + *B. antioquensis* extract 10%; F1	SPF	14.8 ± 2.5 ^a^	8.0 ± 0.7	7.0 ± 0.8	6.0 ± 0.8	6.0 ± 0.7
	UVAPF	7.0 ± 0.5 ^a^	-	-	-	4.0 ± 0.5
	λ_c_	378	379	379	380	380
	UVA/UVB	0.78	0.80	0.80	0.81	0.81
	% SPF_eff_	100.0%	54.1%	47.3%	40.5%	40.5%
	%UVAPF_eff_	100.0%	-	-	-	57.1%
Emulsion + G-E NP (1:1); F2	SPF	17.7 ± 2.2	16.1 ± 2.8	15.6 ± 2.4	15.0 ± 2.3	14.2 ± 2.4
	UVAPF	8.0 ± 0.6 ^b^	-	-	-	7.3 ± 0.4
	λ_c_	379	381	381	382	381
	UVA/UVB	0.79	0.81	0.80	0.80	0.79
	% SPF_eff_	100.0%	91.0%	88.1%	84.7%	80.2%
	%UVAPF_eff_	100.0%	-	-	-	91.3%
Emulsion + L-E NP (1:1); F3	SPF	22.6 ± 2.5	21.8 ± 0.5	21.1 ± 0.3	20.8 ± 0.3	19.9 ± 4.7
	UVAPF	8.7 ± 0.6 ^b^	-	-	-	8.3 ± 0.4
	λ_c_	382	383	383	383	383
	UVA/UVB	0.72	0.73	0.72	0.72	0.72
	% SPF_eff_	100.0%	96.5%	93.4%	92.0%	88.1%
	%UVAPF_eff_	100.0%	-	-	-	95.4%
Emulsion + G-L-E NP (0.5:0.5:1); F4	SPF	13.9 ± 3.7 ^a^	13.7 ± 1.1	13.5 ± 1.0	13.4 ± 1.1	13.2 ± 2.7
	UVAPF	6.0 ± 0.3 ^c^	-	-	-	6.0 ± 0.4
	λ_c_	382	383	383	383	383
	UVA/UVB	0.78	0.79	0.79	0.78	0.77
	% SPF_eff_	100.0%	98.6%	97.1%	96.4%	95.0%
	%UVAPF_eff_	100.0%	-	-	-	100.0%
Emulsion + G-E NP (1:0.5); F5	SPF	9.4 ± 1.4 ^b^	8.8 ± 1.1	8.7 ± 1.0	8.6 ± 1.1	8.5 ± 1.4
	UVAPF	6.0 ± 0.3 ^c^	-	-	-	5.7 ± 0.3
	λ_c_	377	379	379	379	380
	UVA/UVB	0.76	0.76	0.76	0.76	0.76
	% SPF_eff_	100.0%	93.6%	92.6%	91.5%	90.4%
	%UVAPF_eff_	100.0%	-	-	-	95.0%
Emulsion + L-E NP (1:0.5); F6	SPF	14.2 ± 1.7 ^a^	13.0 ± 1.8	12.4 ± 1.9	12.3 ± 1.8	12.3 ± 2.0
	UVAPF	7.3 ± 0.6 ^a^	-	-	-	6.7 ± 0.5
	λ_c_	383	383	383	383	384
	UVA/UVB	0.71	0.72	0.72	0.72	0.72
	% SPF_eff_	100.0%	91.5%	87.3%	86.6%	86.6%
	%UVAPF_eff_	100.0%	-	-	-	91.8%
Emulsion + G-L-E NP (0.5:0.5:0.5); F7	SPF	10.1 ± 0.5 ^b^	10.0 ± 0.4	10.0 ± 0.4	10.0 ± 0.5	10.0 ± 0.1
	UVAPF	5.0 ± 0.6	-	-	-	5.0 ± 0.6
	λ_c_	379	380	380	380	381
	UVA/UVB	0.66	0.66	0.66	0.66	0.66
	% SPF_eff_	100.0%	99.0%	99.0%	99.0%	99.0%
	%UVAPF_eff_	100.0%	-	-	-	100.0%

* CSS: commercial sunscreen. † SPF: sun protection factor. ‡ UVAPF: UVA protection factor. λ_c_: critical wavelength. Results are expressed as the mean value ± standard deviation (*n* = 3). Values in the same column followed by different letters are significantly different at the 5% level.

## Data Availability

The data presented in this study are available in the article.
